# Dietary Selenium Intakes and Musculoskeletal Function in Very Old Adults: Analysis of the Newcastle 85+ Study

**DOI:** 10.3390/nu12072068

**Published:** 2020-07-12

**Authors:** Giorgia Perri, Nuno Mendonça, Carol Jagger, Jennifer Walsh, Richard Eastell, John C. Mathers, Tom R. Hill

**Affiliations:** 1 The MRC-Versus Arthritis Centre for Integrated Research into Musculoskeletal Ageing (CIMA), Newcastle upon Tyne NE2 4HH, UK; j.walsh@sheffield.ac.uk (J.W.); r.eastell@sheffield.ac.uk (R.E.); john.mathers@newcastle.ac.uk (J.C.M.); tom.hill@newcastle.ac.uk (T.R.H.); 2 Human Nutrition Research Centre, Population Health Sciences Institute, Newcastle University, Newcastle upon Tyne NE2 4HH, UK; nuno.mendonca@nms.unl.pt; 3 EpiDoC Unit, NOVA Medical School, Universidade Nova de Lisboa (NMS-UNL), 1150-082 Lisbon, Portugal; 4 Comprehensive Health Research Centre (CHRC), NOVA Medical School, Universidade Nova de Lisboa, 1150-082 Lisbon, Portugal; 5 Population Health Sciences Institute, Campus for Ageing and Vitality, Newcastle University, Newcastle upon Tyne NE4 5PL, UK; carol.jagger@newcastle.ac.uk; 6 Department of Oncology and Metabolism, University of Sheffield, Sheffield S5 7AU, UK

**Keywords:** dietary intake, selenium, very old adults, Newcastle 85+ Study, musculoskeletal function

## Abstract

**Background**: Selenium is a trace element essential for health. Severe selenium deficiencies are associated with poor musculoskeletal (MSK) function. However, the effects of moderate deficiency on MSK function, especially in older adults, is unclear. **Objectives**: To determine the associations between selenium intake and MSK function in very old adults. **Methods**: Selenium intake at baseline and, hand-grip strength (HGS) and timed-up-and-go (TUG) at four phases over 5 years, were available in 791 participants in the Newcastle 85+ Study, a community-based, longitudinal cohort of ≥ 85 year old individuals. We investigated relationships between selenium intake and HGS and TUG in cross-sectional analyses at baseline using multivariate analyses and, prospectively using linear mixed models to explore HGS and TUG changes over 5 years in association with baseline selenium intake. **Results**: At baseline, 53% of participants had selenium intakes that were classified as low. These individuals had 2.80 kg lower HGS and were 2.30 s slower performing the TUG, cross-sectionally. In multivariate, baseline analyses, selenium intake had no significant impact on HGS or TUG. Selenium intake had no significant effect on MSK function, prospectively. **Conclusion**: Low selenium intake is common among very old adults and, in cross-sectional analyses, is associated with poorer MSK function.

## 1. Introduction

### 1.1. Importance of Selenium

Selenium is an essential trace element for normal function of the human body [1,2]. In mammals, selenium is incorporated as selenocysteine (Sec) into 25 characteristed selenoproteins [3]; selenoprotein production relies on dietary selenium and specific cofactors. Sec is encoded by a uracil, guanine, adenine (UGA) codon which requires a Sec-charged transfer ribonucleic acid (tRNA); termination of protein synthesis is prevented due to the presence of the selenocysteine insertion signal (SECIS). Other factors involved are selenophosphate synthetase 2 (SPS2) and an elongation factor (EFsec) [4]. Within the human diet, selenium is obtained from both animal and plant sources [1,5]. The selenium content of crops depends on geographical location which influences soil selenium content and the use of selenium-containing fertilizers [5,6,7,8,9]. Consequently, the selenium intake and selenium status of human populations differs geographically. Lower selenium status occurs widely in Europe, including the UK, New Zealand, Pacific Northeast, and Northeast regions of China, Scandinavia and the South Atlantic Seaboard [5]. Changes in trade practices also influence selenium supply in the human food chain. For example, since the 1980s, when Canadian wheat, which has higher selenium content was replaced by UK-grown wheat with a lower selenium content, the selenium status of the UK population has declined [10,11]. Although the selenium content of bread is not particularly high, the relatively large bread consumption in western countries makes it an important dietary source of selenium [10,11].

### 1.2. Selenium Intakes and Older Adults

Severe selenium deficiency is associated with cardiomyopathy and osteoarthropathy including Keshan and Kashin Beck disease, respectively, which are characterized by joint pain, arthritis, muscle wastage and pain [12,13]. Whilst these severe deficiencies are uncommon in most areas of the world, especially in western countries, selenium inadequacy is a global issue. For example, in the UK, the average selenium intake in adults is 40 μg/d [11] which equates to the lower reference nutrient intake (LRNI) and is considerably below the reference nutrient intake (RNI) for selenium which is 75 and 60 μg/d for men and women over 50 years, respectively [11,14]. This is becoming an even greater risk in older adults (> 65 years) due to lower energy intakes [15] and consequently lower selenium intakes. Furthermore, sufficient energy intakes do not always guarantee adequate selenium intakes; selenium-rich food sources are also protein-rich [5] which can be difficult for older individuals to purchase, prepare or consume [16]. Since older populations are the fastest growing in developed countries it is crucial their nutritional intakes are explored. Undernutrition is a major issue in the ageing population; 14% of UK community-dwelling and 21% of institutionalized adults are at risk [17]. One study found that > 20% of European > 65 years inadequately consumed micronutrients, including selenium [18] and ≥ 30% of non-institutionalized individuals in western countries had vitamin D, B**_2_**, Ca, Mg and selenium intake below the estimated average requirement (EAR) [19]. Poor nutrition, especially lack of micronutrients, including selenium [20,21] has been associated with reduced health, increased oxidative stress and inflammatory markers.

### 1.3. Potential Mechanisms of Selenium Function

Mechanistic studies have shown that selenoproteins, especially those in the glutathione peroxidase family, (GPx) may be important in musculoskeletal (MSK) function by neutralizing reactive oxygen species (ROS) such as hydrogen peroxide [22,23]. Suboptimal selenoprotein levels upregulate inflammatory cytokines [24], leading to muscle weakness and oxidative damage [25]; higher levels of interleukin 6 (IL-6) have been associated with low selenium status [26,27]. IL-6 can impact muscle function and contraction as it interferes with insulin-like growth factor 1 (IGF-1) secretion [28,29,30] and is also involved in bone resorption and receptor activator of nuclear factor kappa-Β ligand (RANK-L) signalling [31,32,33,34]. Likewise, muscle function can be impaired; selenium-deficient patients had elevated levels of serum creatine kinase, muscle fatigue, pain, and proximal weakness [2,35,36,37]. This weakness is seen in other studies where hip, knee and grip strength were poorer in individuals when plasma selenium was in the lowest quartile compared to those in the highest [38]. Similarly, serum selenium has been positively associated with muscle mass [39], physical performance [40], grip strength [41] and negatively associated with sarcopenia prevalence [42,43]. Plasma selenium has also been associated with improved bone mineral density in postmenopausal women [44] and has been found in osteoblasts, suggesting a potential role in bone metabolism [45]. Likewise, selenium-deficient mice were characterized by decreased bone microarchitecture and increased bone resorption and inflammatory biomarkers [46]. Equally, selenium-deficient rats displayed decreased bone health and skeletal growth [47,48,49].

Despite these associations between selenium and MSK function, research is limited [50,51,52] and even fewer studies have explored these associations in the very old, or with adequate sample sizes. Those that have either used self-reported health data, had incomplete follow-ups, poor coverage on health domains or only recruited institutionalized individuals [37,38,39,40,41,42,43]. These issues have been overcome in the Newcastle 85+ study which included individuals born in 1921 regardless of health status. This study was the first, largest, population-based longitudinal study to examine a single year birth cohort in the North East of England exploring health trajectories related to clinical, social and biological factors that prolonged independence and good health.

### 1.4. Aims and Hypotheses

We hypothesised that those with higher selenium intake will have better MSK function at baseline and a slower decline in MSK function over 5 years. We aimed to test this hypothesis by investigating the relationships between dietary selenium intake at baseline and two measures of MSK function, hand-grip strength (HGS) and timed-up-and-go (TUG), at both baseline and 5 year follow-up in very old participants in the Newcastle 85+ Study.

## 2. Materials and Methods

### 2.1. Study Population

Participant data were obtained from the Newcastle 85+ Study, a longitudinal study of health outcomes and trajectories, of 1042 participants born in 1921 who were registered with GPs from North Tyneside and Newcastle upon Tyne primary care trusts in North East England. This cohort was sociodemographically representative of the general UK older population [53]. Dietary exclusions, such as veganism or vegetarianism were not an issue in this study, 94% of participants consumed meat and meat products on the days prior to dietary assessment. The only exclusions were individuals with end-stage terminal illness and those who could not be visited by a lone nurse without posing risks. All individuals provided informed consent, or when this was not possible, consent was provided by an appropriate consultee.

### 2.2. Socioeconomic, Lifestyle and Health Measures

**Figure 1:**
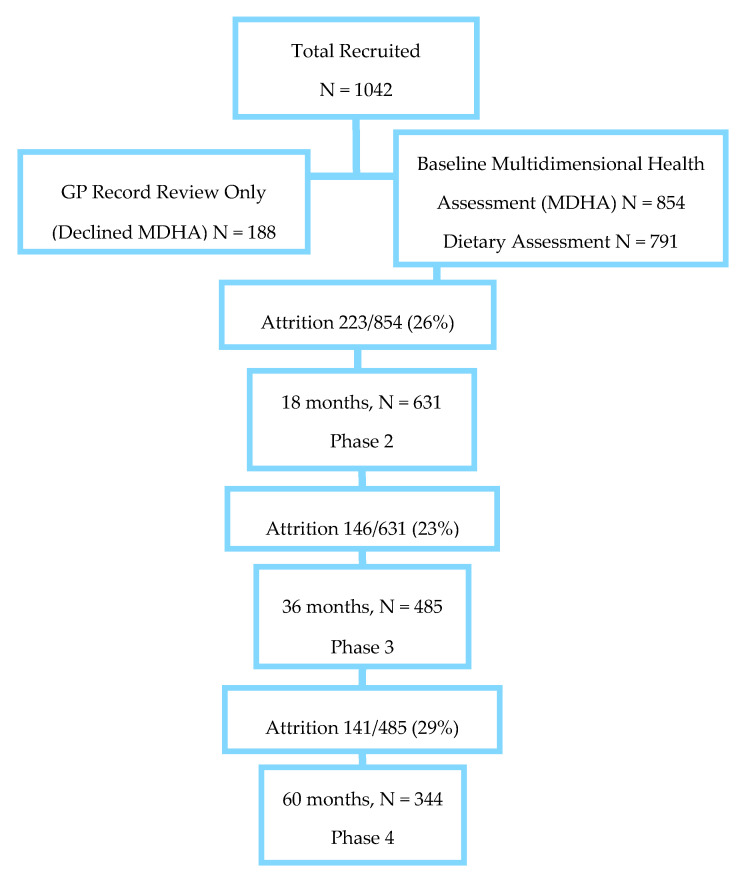
Flow diagram of participant recruitment and sample size at each stage.

Questionnaires, functional tests, fasting blood samples, medical record reviews, dietary intakes and body weight measurements were taken at the initial health assessment (baseline) in 2006/2007 and at three follow-up visits (at 1.5, 3, 5 years, Figure 1). Dietary intake was assessed in 791 participants at baseline only. Individuals had the option to refuse any test. The full study protocol has been published [53] and is also available on the web link (https://research.ncl.ac.uk/85plus/).

The study was conducted in accordance with the Declaration of Helsinki and was approved by the Newcastle and North Tyneside Local Research Ethics Committee 1 (reference number 06/Q0905/2).

Body mass index (BMI) was calculated as kg weight/m^2^ height and categorized as < 18.5 (under-weight)/ > 18.5 < 25 (normal)/ > 25 < 30 (overweight)/30 (obese) [54]. Fat-free mass (kg) was calculated using the Tanita-305 body fat bioimpedance instrument, (Tanita Corp., Tokyo, Japan). Participants were classified into the National Statistics Socio-Economic Classification (NS-SEC) three class scheme [55] based on their previous main occupation. Self-rated health was categorized as excellent/very good, good, fair/poor and cognitive impairment was classified as scores < 25 points on the Standardized Mini-Mental State Examination (SMMSE). Total energy intake (kcal) and protein intake (g) were determined using the 24 h multiple pass recall (MPR). Medication use was determined using GP records. Physical activity was categorized as low/moderate/high (score 0–1/score 2–6/score 7–18, respectively) using a purpose-built questionnaire [56]. Disability score was assessed where 1 indicated difficulty in performance or unable to perform and 0 indicated no difficulty. This was produced by summing the scores of 17 tests: self-reported activities of daily living (ADL), instrumental activities of daily living (IADL), mobility issues, lower limb mobility, chair rises, stair climbing, grocery shopping and walking 370 m, all of which were greatly related to TUG measures [57].

### 2.3. Musculoskeletal Function

HGS was measured [58] using a hand-held dynamometer (Takei A5401). Participants stood with their arm hanging beside their body with their elbow at a 180° angle and squeezed the dynamometer as hard as possible with each hand, in turn. Two measurements (kg) were taken for each hand and the mean of all four measurements was calculated and used in subsequent analyses.

In the TUG test [59,60], participants were asked to rise from a chair (46 cm from the floor with armrests), and as quickly and safely as possible, walk 3 m, turn 180° and return back to the chair and sit down. The time (s) from the first attempt to rise from the chair to when the participant returned and sat on the chair was recorded using a stopwatch. HGS and TUG were measured at baseline, 0 years (Phase 1), 1.5 years (Phase 2), 3 years (Phase 3) and 5 years (Phase 4).

### 2.4. Dietary Assessment

On two separate weekdays (Monday-Thursday, excluding Fridays and weekends) separated by a week, a 24 h MPR was used to assess dietary intake at baseline (2006/2007) in 791 participants (62% females, 38% males) within their usual residence. This technique was retrospective and involved a structured interview to obtain specific information on habitual consumption of food, beverages and supplements over 24 h. A pilot study in the same cohort found that this method was more reliable than a food frequency questionnaire (FFQ) [61]. To estimate food and drink portions, the Photographic Atlas of Food Portion Sizes [62] was used and data was entered twice, independently to reduce errors. The McCance and Widdowson’s composition of Food [63] was used alongside a Microsoft Office Access database containing nutrient compositions of frequently consumed foods to predict energy, macronutrient and micronutrient intakes using the 2-day mean intakes [64]. Most participants (85%) revealed that they felt the 24 h MPR replicated their habitual intakes of food and drink [65], which was assessed using a questionnaire following the 24 h MPR Across all dietary assessments, misreporting is a limitation. Using cut-off values derived from energy intake (EI) estimations divided by estimated basal metabolic rates (BMR_est_) (EI:BMR**_est_**) is one way to detect misreporters [66]. In older participants, a more accurate technique is the Fredrix equation, which was used in this study [64]; EI:BMR_est_ < 1.05 and < 2.0 indicate under and over reporters, respectively [67].

Supplement data was obtained from individuals and was created as a binary variable: 0, 1 indicating no supplementation use and supplement use, respectively. A binary variable was used instead of continuous variable to reduce inaccuracy as there was a limitation in the information provided from participants regarding the exact concentrations and doses. However, this variable was not included in the reported analyses due to uneven representations (2.4% of the population consumed selenium-containing supplements).

Selenium (µg) was estimated as a daily intake; values were assessed to determine which individuals consumed below the LRNI (40 µg/d), between the LRNI and up to the RNI (60 µg/d for females and 75 µg/d for males) and the RNI and above. Participants with selenium intakes below the LRNI were suggested to be inadequate since this level of nutrient only meets the needs of 2.5% of a specified group.

Individual foods were categorized into fifteen first-level groups (Table 1). These groups were used to define the percentage contribution of the total selenium intake and determine which groups contributed the greatest to selenium intakes across all participants subcategorized by males and females.

### 2.5. Statistical Analyses

IBM statistical software package version 24.0 (SPSS) was used to perform the exploratory and statistical analyses, *p* < 0.05 was considered statistically significant. To determine normality of the variables, the Shapiro-Wilk test and quantile–quantile (QQ) plots were used. Selenium intake was categorized into biologically relevant tertiles (low < 40, moderate 40–59 (women), 40–74 (men) and optimal, ≥ 60 (women), ≥ 75 (men) µg/d). These cut-offs were less severe compared to binary variables (above and below LRNI) and due to the large sample size there was an adequate split across the groups (low, moderate and optimal, n = 417, 261 and 113, respectively) which provided a biologically-relevant stance to help implement current dietary guidelines.

Descriptive statistics were used to determine baseline characteristics and the percentage of individuals within each intake group (low, moderate, and optimal). Differences in characteristics across intake groups were assessed using student t-test or one-way ANOVA (normally distributed), Chi-square test (categorical) and Kruskal–Wallis (ordered and non-normally distributed). The latter test was used most frequently as the majority of data were non-normally distributed.

To explore the selenium intakes further, food groups were analysed to determine those that contributed greatest to selenium intake. First level food groups (Table 1) were aggregated so intakes from the same group with the same value were summed.

To determine the top contributors of selenium to total selenium intake the equation: (selenium from selected food/total selenium intake) × 100 was used. The top > 90% contributors were plotted, and the remaining groups were compiled and referred to as “others”. Results are not shown.

HGS and TUG means were compared by selenium intake categories (low, moderate and optimal) using a one-way ANOVA for HGS values and Kruskal–Wallis for non-transformed TUG values at baseline and follow-up. The presence of hand arthritis and use of walking aids were also compared over time and across the different intake categories. Multivariate analyses were used with MSK measures (HGS and TUG) as dependent variables with categorical selenium intake (low, moderate, optimal) as the independent variable, in addition to: age at baseline (continuous), sex (men/women, binary), NS-SEC (routine/manual, intermediate, managerial/professional occupations, categorical), self-rated health (excellent/very good, good, fair/poor, ordinal), energy intake (continuous), protein intake (continuous), medication use (continuous), BMI (under-weight, normal, obese, ordered), fat-free mass (continuous), physical activity (low/moderate/high, ordinal), cognitive impairment (continuous), disability score (categorical), misreporters (binary) and change in diet (binary). A spearman rank-order correlation was also performed between selenium (low, moderate, optimal) and protein and energy intakes. HGS values were normally distributed, however TUG values were positively skewed and were therefore log transformed and used as continuous variables for prospective analyses.

Linear mixed models were used to determine the association between dietary selenium categories and initial level and rate of change in baseline HGS and TUG over 5 years in all participants, men and women. Time was treated as a categorical variable for each phase (1–4). Time and the intercept were used as random effects. Fixed effects were the selected variables of interest. We used three different models: (1) time and selenium; (2) time and selenium interactions [time x selenium]; (3) adjustments made for presence of hand arthritis or use of walking aids (binary), age at baseline, sex, NS-SEC, self-rated health, energy intake, protein intake, medication use, BMI, fat-free mass, physical activity, cognitive impairment, disability score, misreporters and change in diet. These covariates were selected based on previous research using the same cohort in the effects of vitamin D and protein on MSK function [68,69,70]. Restricted maximum likelihood (RML) and unstructured or heterogeneous first-order autoregressive covariance matrixes were applied to derive parameter estimates (β). Negative and positive β estimates for HGS and TUG, respectively, indicated poorer performance. Graphical outputs were created in Microsoft Excel 2010 using the equation: Intercept value + Time × (Time-beta + Time × selenium-beta interaction term) + selenium-beta.

### 2.6. Sensitivity Analysis

Analyses were repeated for the linear mixed model, using follow-ups from baseline to 3 years (0, 1.5, 3 years) to maintain the same interval of increase in age throughout prospective analyses. The adjusted model (Model 3) used the same covariates.

## 3. Results

### 3.1. Participant Characteristics

Baseline characteristics of 791 participants (76.8%) grouped by selenium intake (low < 40, moderate 40–59 (women), 40–74 (men) and optimal ≥ 60 (women), ≥75 (men) µg/d) are shown in Table 2. The median selenium intakes were 39, 48 and 35 µg/d in all participants, males and females, respectively. Most individuals (52.7%, n = 417) had low intakes consuming < 40 µg/d whilst 14.3% (n = 113) consumed an optimal amount. There was a significant difference between the median intakes in each group (*p* < 0.001), which were 27, 51 and 88 μg/d for low, moderate and optimal intakes, respectively.

The main differences between groups were sex; women were more likely to be in the low selenium group compared to males (72.9 vs. 27.1%, *p* < 0.001). Similarly, total energy (1492 vs. 1989 kcal) and macronutrient intakes were significantly lower in the low selenium group compared to the optimal intake group (fat, carbohydrates and protein 35, 26 and 59% increase, respectively *p* < 0.001). Free triiodothyronine (T3) (pmol/L) was significantly lower in the low selenium group (4.5 vs. 4.6 pmol/L *p* = 0.002), whilst misreporting food intakes were higher in the lowest group compared to those with optimal intakes (40% higher, *p* < 0.001). Other significant differences across selenium groups were smoking, use of walking aids (22% difference), HGS (19% difference), TUG (17% difference), and disability score (61% difference in those with no disability) (Table 2).

### 3.2. Food Intakes of Selenium

Top food group contributors for the same cohort have been previously stated and are therefore, not reported here [65]. In summary, across all individuals the top contributors were “cereals and cereal products”, “meat and meat products”, “fish and fish dishes”, “milk and milk products”, “eggs and egg dishes” and “potatoes” (data not shown). Each food group differed significantly in contribution to total selenium intakes (*p* < 0.005). There was a strong correlation between selenium (low, moderate, optimal), and protein (*r_s_* = 0.503, *p* < 0.001) and total energy intake (*r_s =_* 0.395, *p* < 0.001) (data not shown).

### 3.3. Musculoskeletal Performance

#### 3.3.1. Cross-Sectional Analyses

**Figure 2:**
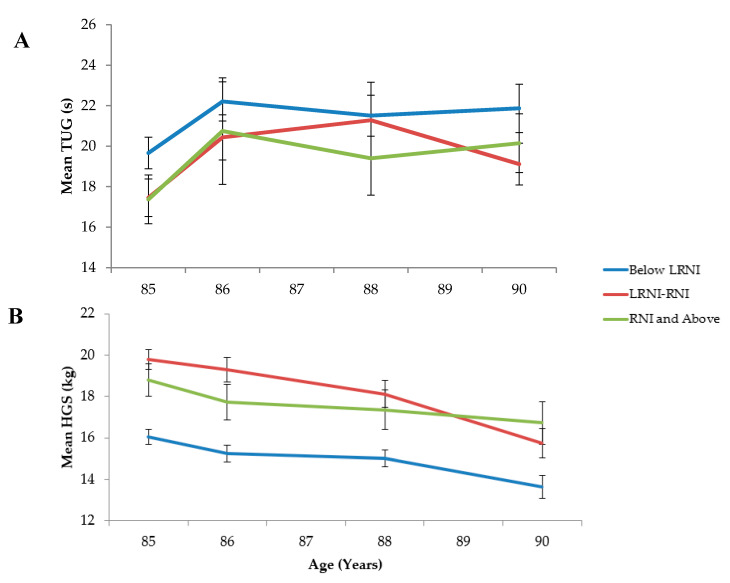
Mean hand grip strength (HGS) (**A**) and timed-up-and-go (TUG) (**B**) values across 5 years divided by selenium intakes: below lower reference nutrient intake (LRNI) (below 40 µg/d), LRNI-reference nutrient intake (RNI) (between 40 and 59 and 40 and 74 µg/d for women and men, respectively) and RNI and above (≥ 60 and ≥ 75 µg/d for women and men, respectively). Time intervals between each measurement were not consistent: 0, 1.5, 3 and 5 years.

HGS and TUG by selenium intakes at baseline and follow-up are shown in Table 3 and Figure 2. Within HGS, there was a significant difference between selenium intakes, where the lowest intakes coincided with the lowest values (baseline: lowest 16.0 ± 7.1, highest 18.8 ± 8.2 *p* < 0.001). The presence of hand arthritis at baseline was not significantly different across groups (*p* = 0.202). Lower selenium intakes were associated with increased TUG; this was significant only at baseline (lowest 19.7 ± 15.4, highest 17.4 ± 12.3 *p* = 0.009) (Figure 2, Table 3). The use of walking aids was significantly different across intake groups at baseline only (*p* = 0.046). In multivariate analyses, selenium intake did not have a significant impact on either HGS or TUG after adjusting for covariates (*p* > 0.05) (data not shown). 

#### 3.3.2. Prospective Analyses

##### Hand Grip Strength

**Figure 3:**
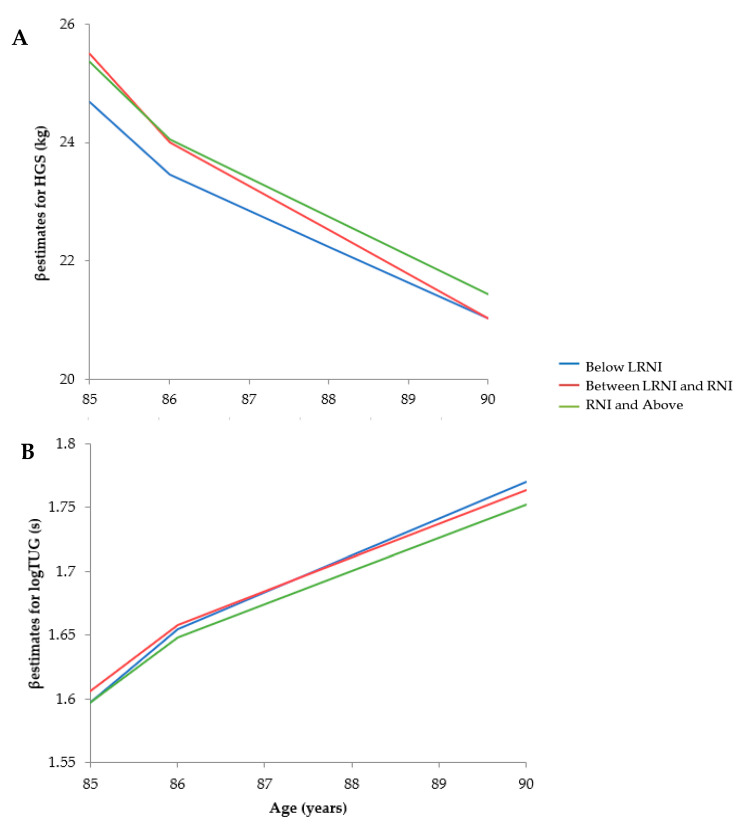
Linear slopes for musculoskeletal measures over time with the presence of hand arthritis (hand grip strength, HGS, **A**) or the use of walking aids (timed-up-and-go, TUG, **B**) as predictors in addition to age at baseline, sex, National Statistics Socio-Economic Classification (NS-SEC), self-rated health, energy intake, protein intake, medication use, body mass index (BMI), fat-free mass, physical activity, cognitive impairment, disability score, misreporters and change in diet. Time intervals between each measurement were not consistent: 0, 1.5, 3 and 5 years.

Associations between selenium intakes and HGS change over 5 years are shown in Table 4, Supplementary Material Table S1 and Figure 3. Time had a significant impact on HGS leading to a decline in strength in all individuals, men and women (*p* < 0.001). In Model 1 (unadjusted), there was an overall −1.25 kg loss in HGS across all individuals over time; this was greater in men with a −1.76 kg loss compared to women with a −0.92 kg loss (Supplementary Material Table S1). Low selenium intakes had a significant effect on HGS in all individuals (β −2.70 ± 0.76, *p* < 0.001) in Model 1 and Model 2 (β −2.94 ± 0.88, *p* < 0.001), but not in males or females when analysed separately (Supplementary Material, Table S1). In the fully adjusted model (Model 3, Table 4), low selenium intake did not have a significant impact (*p* = 0.292), however, a lack of hand arthritis was a significant, positive predictor of baseline HGS (β 3.69 ± 0.745, *p* < 0.001, data not shown).

##### Timed Up and Go

Over the 5 years, time had a significant effect on TUG (log10-transformed) performance indicating a 0.054 log10-s increase in the time for participants to rise from a chair (*p* < 0.001); TUG performance increased by 0.058 log_10_-s and 0.052 log_10_-s for men and women, respectively (Supplementary Material, Table S1 continued). Low selenium intake had a significant effect on TUG in all individuals (β 0.049 ± 0.025, *p* = 0.048) in Model 1, but not for males or females when analysed separately (Supplementary Material, Table S1).

In the fully adjusted model (Model 3, Table 4), lower selenium intake did not have a significant impact on TUG (*p* = 0.364), however walking aids were a significant predictor of baseline TUG (β −0.191 ± 0.017, *p* < 0.001, data not shown).

### 3.4. Sensitivity Analysis

Compared to the results using four time points (0, 1.5, 3, 5 years), there were no significant differences in the effect of selenium intake on either HGS or TUG when using three time points (data not shown).

## 4. Discussion

### 4.1. Summary

Median intakes of selenium were below the RNI (38.9 ± 28.2 µg/d ) in all participants. Intakes below the LRNI occurred in 53% of participants; these were more frequent in women, smokers, those with lower free T3, those that used walking aids and had a higher disability score. The top food contributors for all consumers were cereals, meat, fish, eggs, milk and potatoes. Those with the lowest intakes had 2.80 kg lower HGS and 2.30 s slower TUG at baseline compared to those with higher intakes. There was no significant effect of selenium intake on HGS or TUG in multivariate analyses at baseline or over time in prospective analyses. However, time had a significant effect on the rate of change over 5 years in both parameters.

### 4.2. Selenium Intakes

Many studies have reported inadequate selenium intakes; approximately 76% of women and 39% of men aged 75 years and above had selenium intakes below the LRNI [71]. Similarly, in Northern Ireland, 73% of participants with chronic heart failure failed to meet the RNI [72]. Inadequate intakes are also seen outside of the UK; in a cross-sectional study using New Zealand women, 83% were 2/3rd below the Australian RNI (70 μg/d) [73]. However, the UK EPIC-Oxford Study [74] found higher selenium intakes compared to our results and many others, although age ranges were larger (≤ 80 years) and the statistics, dietary assessment and participant characteristics differed which could potentially explain the differences [75]. Health declines including intakes are not linear with age; advanced ageing leads to more inter-variability and quicker declines [76]; prevalence of successful ageing in American individuals ≥85 years compared to 65–74 years and 75-84 years was 16.2% and 6.5% times lower [77] and equally, healthy ageing decreased with increasing age in European adults [78]. Our study reduces this heterogeneity by using a single-year birth cohort.

### 4.3. Sources of Selenium

Consistent with our results, the most common selenium-containing foods for older adults are cereals, followed by animal sources, rather than vegetables and legumes [15,79,80]. In older adults (> 71 years), 60% of protein was obtained from animal sources such as diary, beef, poultry, pork, fish and eggs [81]. Many of these animal proteins also contain additional nutrients, for example pork contains selenium, phosphorus, potassium and B-vitamins [82]; this was seen in another study where meat and fish eaters had significantly higher selenium intakes than vegans or vegetarians [83]. Similarly, cereals, fish, meat and dairy were major selenium contributors in another study [15], although selenium intakes and serum concentrations were within adequate ranges. These differences may be due to different food composition tables, food origin, protein source or age of the participant [84].

Fish was the 3rd highest selenium contributor in our results [65]; likewise, in another study adding 1 portion of fatty fish increased HGS by 0.43 kg and 0.48 kg in men and women, respectively [85]. Other studies have also found higher HGS with consumption of fatty fish, wholegrains, fruit and vegetables [86,87,88]. Fish therefore appears to be an important factor for HGS, potentially due to its antioxidant properties and protein content. Oxidative protein damage was associated with a lower HGS [89] and physical performance [90]. Improvements in walking were seen in women with higher selenium, vitamin D, β-carotene and protein intakes [40]. In our study, the association between selenium intakes seemed to be weaker for TUG, this could be explained by the fact that the most abundant store of selenium is within muscle and HGS is a good proxy for muscle strength whilst TUG tests are more complex, requiring balance, cognition and motor control [91]. This lack of significance was also seen in another study when dietary selenium data was used [73], although this study excluded institutionalized individuals, had a small sample size and used self-administered FFQ. However, there was a positive association with serum selenium and physical performance [41,73] which was also seen in New Zealand adults (65 years) [92] with higher toenail selenium concentrations.

### 4.4. Potential Mechanistic Roles of Selenium

A hypothesized mechanism for the effect of selenium on MSK function is that antioxidants, including the selenoprotein family, GPx [93,94,95] play a role in muscle function [96,97] by being protective against ROS [98], and therefore, maintain function [99,100]. Oxidative damage increases with age due to a decrease in the neutralization of ROS and reactive nitrogen species (RNS) from a lower intake of antioxidants [101,102]. Large quantities of oxygen are required by skeletal muscle which then produces RNS; excessive accumulation of RNS coincides with a loss of muscle strength and mass by increasing protein breakdown and reducing muscle protein synthesis [99,103]. Since GPx can neutralize RNS and ROS, it could play a potential role in maintaining muscle function. Other factors that play a role in reduced muscle function are decreases in neuromuscular junction capacity [104] or a change in body composition leading to a loss of lean tissue which reduces energy storage capacities, mobility and metabolism regulation [105]. Decreased muscular function is linked to poor nutrition. Likewise, poor muscular performance leads to reduced activity which often reduces appetite [106], energy intake and therefore antioxidant consumption [107]. A reduction in antioxidants, including selenium, has been linked to suboptimal selenoprotein expression [108] and therefore reduced protection against oxidative damage [109,110].

### 4.5. Strengths and Limitations

There were more women than men and a low ethnic diversity in this study population. This was a potential limitation due to increased disease in very old adults and differences in disease risks across ethnicities. Further bias could be introduced due to the capacity of older adults to partake in assessments leading to incomplete data sets, although this is common across all studies using older populations. Complete baseline HGS measures came from a greater percentage of men, who were well-educated with greater physical activity levels. In addition, due to the age range, attrition and mortality were high [111]; this may have introduced bias to healthier survivors, especially in males where cognitive decline, depression and diseases were lower [111]. This, along with selenium dietary data limited to baseline, could explain the lack of severe decline in muscle performance over time (Figure 3). Other influential factors (frailty and dietary knowledge) [112] which potentially affected baseline selenium levels in weaker individuals were not accounted for, however, adding more covariates to the adjusted model may have reduced statistical power.

There were some limitations in using the 24 h MPR, for example only measuring the diet at baseline on two non-consecutive days (excluding Friday and Saturday) may not reflect consumption of fish, a good selenium contributor in this cohort and others [65], which is traditionally consumed on Friday in Britain. These changes in diet over the weekend may lead to less robust measures, although, it is unclear how weekends affect dietary intakes in the elderly and whether intake remains stable over 5 years [113,114]. More recalls were made in summer (35%) with the rest evenly divided across the remaining three seasons [65]. Micronutrient intakes can be altered by seasonality, although the slight bias in summer recordings is unlikely to change the results and selenium content is less likely to be seasonally affected, as seen in milk [115]. Older individuals may have struggled with the retrospective method leading to underestimation of snack foods, although these had a minimal contribution to selenium intakes (< 0.01%). As with all nutritional assessments, there are limitations in using food composition tables to determine selenium intakes [116]; data can be non-representative of the foods consumed, for example, cooking methods can alter selenium concentrations by up to 40% [117] and concentrations can differ within food type (Italian pasta contained 51 ug/100g less selenium than US pasta) [118]. Likewise, data can be incomplete where selenium values are absent and unknown despite being a selenium-dense food. In the McCance and Widdowson 6th Edition [63], 56% of food sources were reported to contain selenium but lacked values (referred to as “N”), and 80% of these came from Meat and Meat Products leading to a large misrepresentation. Using standardized cooking methods and food sources [119] and re-analyzing selenium content of food may help dissipate this issue, although, a more reliable, albeit expensive and invasive technique would be analyzing selenium status.

Many studies have used selenium status (plasma or serum) rather than dietary intakes. For example, hair and serum selenium levels were adequate in home-living postmenopausal women from high socioeconomic backgrounds when consuming their habitual diets [120]. Plasma and serum selenium measures appear to be better indicators of selenium status than dietary intakes and overcome the issues associated with food composition databases [116].

As with all observational studies, causation between dietary selenium intake and muscle function cannot be implied. Randomised, placebo-controlled trials would be helpful in determining the associations between moderate selenium deficiency and MSK outcomes; these studies are scarce or lack an adequate number of participants. One study using selenium-deficient participants with either an intravenous sodium selenite supplementation (200 μg, 5–7/wk) or a placebo for 4 months, found that supplementation improved serum selenium and mean diameter of type 1 muscle fibres, but did not improve quadriceps strength, however, this trial only used 10 participants [121]. Trials like this could be repeated with a larger sample size and different supplementation methods or forms of selenium to further elucidate the roles of selenium in muscle strength and function.

The strengths of the Newcastle 85+ study are its large sample size where individuals living in all sectors were included without health discrimination. Detailed information on participant health was obtained from medical records rather than self-reported data, which can be less reliable in elderly participants due to cognitive decline and increased comorbidities. Other strengths were multilevel analyses of stratified sub cohorts (by sex and selenium categories) and being a prospective study using a single birth cohort (born 1921) with a homogenous age. The study population was sociodemographically representative of the local population when compared to the 2001 National Census data with regards to the proportions living in care and those with cognitive impairment and used a stable population with similar ethnicity [53]. Selenium variability within foods was also reduced since all participants were based in the North East.

## 5. Conclusions

Overall, these results show that inadequate dietary selenium intakes were common in very old adults. Low selenium intakes were associated with poorer HGS and TUG performance in the cross-sectional analyses and in the unadjusted prospective analyses in all individuals, but not after accounting for other covariates. This is likely due to the limited dietary assessments only available at baseline or the proxy measure of selenium status using intakes rather than serum or plasma selenium. Future studies could measure selenium status as well as intake and continue these throughout the study duration.

## Figures and Tables

**Table 1:** List of fifteen first-level food groups used for dietary assessment and analyses.

	Food Group
**1**	cereal and cereal products
**2**	milk and milk products
**3**	eggs and egg dishes
**4**	oils and fat spreads
**5**	meat and meat products
**6**	fish and fish dishes
**7**	vegetables
**8**	potatoes
**9**	savoury snacks
**10**	nuts and seeds
**11**	fruits
**12**	sugar, preserves and confectionery
**13**	non-alcoholic beverages
**14**	alcoholic beverages
**15**	miscellaneous

**Table 2:** Characteristics of study participants by selenium intake groups (low < 40 µg/d, moderate 40–59, 40–74 µg/d, good ≥ 60, ≥ 75 µg/d for women and men, respectively) at baseline.

Characteristic	All Participants	Low Se (< 40 µg/d)	Moderate Se (40–59, 40–74 µg/d)	Optimal Se (≥ 60, ≥ 75 µg/d)	*p*
*Socio-demographic factors*	n = 791	n = 417	n = 261	n = 113	
Women % (n)	61.8 (489)	72.9 (304)	46.7 (122)	55.7 (63)	< 0.001 ^a^
Men % (n)	38.1 (302)	27.1 (113)	53.5 (139)	44.2 (50)	
Years of education % (n)					0.838 ^c^
0–9	64.1 (501)	65.4 (270)	64.1 (164)	59.8 (67)	
10–11	23.4 (183)	22.3 (92)	24.2 (62)	25.9 (29)	
≥ 12	12.4 (97)	12.3 (51)	11.7 (30)	14.3 (16)	
Occupational class % (n)					0.427 ^c^
Managerial and Professional	26.2 (270)	33.3 (139)	31.8 (83)	32.7 (37)	
Intermediate	11.0 (113)	13.9 (58)	11.5 (30)	18.6 (21)	
Routine and Manual	40.3 (415)	47.2 (197)	52.1 (136)	46.0 (52)	
Living in Institution					0.149 ^c^
Yes	8.7 (69)	7.4 (31)	8.8 (23)	13.3 (15)	
No	91.3 (722)	92.6 (386)	91.2 (238)	86.7 (98)	
*Diet-related factors*					
Diet change in past year % (n)					0.502 ^c^
Yes	6.9 (53)	5.9 (24)	8.0 (20)	8.1 (9)	
No	93.1 (718)	94.1 (385)	92.0 (231)	91.9 (102)	
Selenium μg/d (Median, IQR)	39.5, 29.7	27.3, 13.9	51.4, 12.6	87.5, 31.2	< 0.001 ^a^
Total energy kCal (M, SD)	1678.6, 507.6	1492.9, 455.0	1841.5, 463.2	1987.9, 513.9	< 0.001 ^a^
Carbohydrate	201.5, 63.2	181.9, 58.3	218.6, 60.9	228.6, 63.4	< 0.001 ^a^
Fat	69.5, 26.6	61.9, 24.0	74.7, 25.4	83.4, 29.3	< 0.001 ^a^
Protein	64.9, 22.6	53.2, 15.9	72.1, 20.6	84.4, 26.5	< 0.001 ^a^
Misreporting food intake ^b^ % (n)					< 0.001 ^c^
Yes	17.0 (124)	23.5 (89)	8.1 (20)	14.3 (15)	
No	83.0 (606)	76.5 (290)	91.9 (226)	85.7 (90)	
*Lifestyle factors*					
Smoking % (n)					0.033 ^c^
Non-Smoker	94.3 (745)	94.5 (393)	96.2 (251)	89.4 (101)	
Current Smoker	5.7 (45)	5.5 (23)	3.8 (10)	10.6 (12)	
Current alcohol intake % (n)					0.712 ^c^
Yes	70.9 (380)	70.4 (197)	73.0 (127)	68.3 (56)	
No	29.1 (156)	29.6 (83)	27.0 (47)	31.7 (26)	
Physical activity (PA) % (n)					0.398 ^c^
Low (score 0–1)	22.1 (176)	24.0 (100)	19.5 (51)	22.1 (25)	
Moderate (score 2–6)	40.7 (343)	44.7 (186)	42.7 (111)	40.7 (46)	
High (score 7–18)	37.2 (270)	31.3 (130)	37.7 (98)	37.2 (42)	
Selenium Supplement Use					0.860 ^c^
Yes	3.2 (25)	3.4 (14)	2.7 (7)	3.5 (4)	
No	96.8 (766)	96.6 (403)	97.3 (254)	96.5 (109)	
*Health-related factors*					
Self-rated health					0.270 ^c^
Excellent/Very Good	32.0 (330)	37.6 (157)	44.1 (115)	40.7 (46)	
Good	30.1 (310)	35.7 (149)	35.2 (92)	40.7 (46)	
Fair/Poor	17.9 (184)	24.2 (101)	19.2 (50)	17.7 (20)	
Disability Score % (n) n = 785					0.036^c^
No Disability	20.5 (161)	16.7 (69)	23.8 (62)	27.0 (30)	
Low (score 1–6)	52.1 (409)	54.7 (226)	51.0 (133)	45.0 (50)	
Moderate (score 7–12)	18.6 (146)	17.7 (73)	19.5 (51)	19.8 (22)	
High (score 13–17)	8.8 (69)	10.9 (45)	5.7 (15)	8.1 (9)	
GDS score (M, SD)	3.3, 2.4	3.3, 2.3	3.3, 2.3	3.4, 2.6	0.713 ^a^
Cognitive Impairment (M, SD)					0.948 ^a^
Normal (26–30 SMMSE score)	27.3, 2.7	27.3, 2.8	27.3, 2.6	27.4, 2.6	
Free T4 in pmol/L (M, SD)	15.6, 2.6	15.7, 2.7	15.5, 2.4	15.8, 2.9	0.778 ^a^
Free T3 in pmol/L (M, SD)	4.6, 0.5	4.5, 0.5	4.6, 0.6	4.6, 0.5	0.002 ^a^
Phase 1 Arthritis in hands % (n)					0.202 ^c^
Yes	7.1 (55)	8.6 (35)	5.9 (15)	4.5 (5)	
No	92.9 (719)	91.4 (371)	94.1 (241)	95.5 (107)	
Phase 1 Walking Aids % (n)					0.046 ^c^
Yes	17.8 (131)	20.9 (81)	13.2 (32)	17.1 (18)	
No	82.2 (604)	79.1 (306)	86.8 (211)	82.9 (87)	
HGS Phase 1 (M, SD)	18.7, 7.7	16.8, 7.2	20.8, 7.5	20.0, 7.8	< 0.001 ^b^
TUG Phase 1 (M, SD)	17.3, 13.1	18.3, 13.5	16.7, 14.2	15.1, 6.8	0.009 ^a^
*Anthropometry*					
BMI (M, SD)	24.5, 4.4	24.6, 4.7	24.6, 4.0	24.0, 4.0	0.636 ^a^

a: Kruskal–Wallis test for ordered and non-normally distributed continuous variables. b: One-way ANOVA for normally distributed data. c: χ^2^ test was used for all other categorical variables. TUG: timed up and go. HGS: hand grip strength.

**Table 3:** Untransformed hand grip strength and timed up-and-go scores by selenium intake groups at baseline and follow-up.

Physical Performance	All Participants	Low Se	Moderate Se	Optimal Se	*p*
	**Hand Grip Strength**				
Baseline (n)	813	401	254	112	
kg (M, SD)	17.5, 7.7	16.0, 7.1	19.8, 7.8	18.8, 8.2	< 0.001 ^a^
Hand arthritis, % (n) yes	0.1 (55)	63.6 (35)	27.3 (15)	0.1 (5)	0.202 ^b^
Follow-up at 1.5 years (n)	605	306	198	90	
kg (M, SD)	16.9, 7.8	15.3, 6.9	19.3, 8.3	17.7, 8.2	< 0.001 ^a^
Follow-up at 3 years (n)	452	226	148	71	
kg (M, SD)	16.4, 7.3	15.0, 6.3	18.1, 7.9	17.4, 7.9	< 0.001 ^a^
Follow-up at 5 years (n)	294	140	97	54	
kg (M, SD)	14.9, 7.0	13.7, 6.5	15.7, 7.1	16.7, 7.6	0.008 ^a^
	**Timed Up-and-Go**				
Baseline (n)	747	387	243	105	
s (M, SD)	18.7, 14.7	19.7, 15.4	17.5, 14.5	17.4, 12.3	0.009 ^c^
Use of walking aids, % (n) yes	17.8 (131)	61.8 (81)	24.4 (32)	13.7 (18)	0.046 ^b^
Follow-up at 1.5 years (n)	547	277	184	80	
s (M, SD)	21.4, 17.1	22.2, 16.1	20.4, 15.1	20.7, 23.6	0.095 ^c^
Use of walking aids, % (n) yes	16.6 (90)	56.7 (51)	27.8 (25)	15.6 (14)	0.407 ^b^
Follow-up at 3 years (n)	402	199	134	63	0.098 ^c^
s (M, SD)	21.5, 18.8	21.5, 14.2	21.3, 21.8	19.4, 14.5	
Use of walking aids, % (n) yes	17.7 (70)	57.1 (40)	30.3 (21)	12.9 (9)	0.421 ^b^
Follow-up at 5 years (n)	274	134	86	49	
s (M, SD)	20.8, 12.2	21.9, 13.8	19.1, 9.7	20.1, 10.2	0.529 ^c^
Use of walking aids, % (n) yes	26.1 (71)	49.3 (35)	29.6 (21)	21.1 (15)	0.733 ^b^

a: Student t-test for normally distributed continuous variables. b: χ^2^ test for categorical variables. c: Kruskal–Wallis test for non-normally distributed continuous data (untransformed).

**Table 4:** Hand grip strength (kg) and timed-up-and-go (log_10_-s) trajectory estimates in low, moderate and optimal selenium over 5 years separated by sex.

Outcome	Variable	Model 3	
		β (SE)	*p*
HGS (kg)	Intercept	25.36 (33.11)	0.444
ALL INDIVIDUALS	Se intake group		
	Low Se	−0.69 (0.65)	0.292
	Moderate Se	0.14 (0.64)	0.829
	Decline		
	Time	−1.31 (0.17)	< 0.001
	Slopes (rate of decline)		
	Se intake × Time		
	Low Se × Time	0.09 (0.20)	0.650
	Moderate Se	−0.18 (0.21)	0.387
HGS (kg)	Intercept	125.81 (59.65)	0.036
MEN	Se intake group		
	Low Se	1.19 (1.19)	0.317
	Moderate Se	0.94 (1.07)	0.384
	Decline		
	Time	−1.69 (0.31)	< 0.001
	Slopes (rate of decline)		
	Se intake × Time		
	Low Se × Time	−0.25 (0.39)	0.513
	Moderate Se	−0.16 (0.37)	0.669
HGS (kg)	Intercept	−23.82 (34.80)	0.045
WOMEN	Se intake group		
	Low Se	−0.91 (0.69)	0.184
	Moderate Se	−0.08 (0.73)	0.915
	Decline		
	Time	−1.05 (0.18)	< 0.001
	Slopes (rate of decline)		
	Se intake × Time		
	Low Se × Time	0.10 (0.21)	0.626
	Moderate Se	−0.05 (0.23)	0.826
TUG (log_10_-s)	Intercept	1.65 (1.08)	0.126
ALL INDIVIDUALS	Se intake group		
	Low Se	−0.002 (0.024)	0.364
	Moderate Se	−0.009 (0.024)	0.722
	Decline		
	Time	0.051 (0.010)	< 0.001
	Slopes (rate of decline)		
	Se intake × Time		
	Low Se × Time	0.020 (0.012)	0.091
	Moderate Se	0.013 (0.013)	0.301
TUG (log_10_-s)	Intercept	−2.949 (3.239)	0.365
MEN	Se intake group		
	Low Se	−0.016 (0.045)	0.730
	Moderate Se	0.031(0.041)	0.457
	Decline		
	Time	0.054 (0.013)	< 0.001
	Slopes (rate of decline)		
	Se intake × Time		
	Low Se × Time	0.002 (0.016)	0.917
	Moderate Se	−0.010 (0.015)	0.507
TUG (log_10_-s)	Intercept	0.629 (2.305)	0.785
WOMEN	Se intake group		
	Low Se	−0.023 (0.037)	0.541
	Moderate Se	−0.029 (0.039)	0.462
	Decline		
	Time	0.042 (0.010)	< 0.001
	Slopes (rate of decline)		
	Se intake × Time		
	Low Se × Time	0.006 (0.011)	0.577
	Moderate Se	0.003 (0.012)	0.801

SE, standard error, selenium intakes were divided by below LRNI (< 40 µg/d) and moderate intakes (41–59, 41–74 µg/d in females and males, respectively). Adequate selenium intakes were used a reference. Model 3 is adjusted for presence of hand arthritis or use of walking aids, age at baseline, sex, National Statistics Socio-Economic Classification (NS-SEC), self-rated health, energy intake, protein intake, medication use, body mass index (BMI), fat-free mass physical activity, cognitive impairment, disability score, misreporters, change in diet. Estimated β coefficients (SE) using HGS and TUG longitudinal data.

## Supplementary Materials

The following are available online at https://www.mdpi.com/2072-6643/12/7/2068/s1, Table S1: hand grip strength (kg) and timed-up-and-go (log_10_-s) trajectory estimates in low, moderate and optimal selenium over 5 years separated by sex.
