# Supplementary material for: Dietary Selenium Intakes and Musculoskeletal Function in Very Old Adults: Analysis of the Newcastle 85+ Study
Source: Nutrients. 2020 Jul 12;12(7):2068. doi: 10.3390/nu12072068 (PMC7400825; doi:10.3390/nu12072068)
Supplement: Supplementary file 1 [file nutrients-12-02068-s001.pdf]

Table S1: Hand grip strength (kg) and timed-up-and-go (log_{10}-s) trajectory estimates in low, moderate and optimal selenium over 5 years separated by sex.

Outcome	Variable	Model 1		Model 2		Model 3	
		**β (SE)**	***p***	**β (SE)**	***p***	**β (SE)**	***p***
HGS (kg)	Intercept	20.06 (0.69)	< 0.001	20.05 (0.78)	< 0.001	25.36 (33.11)	0.444
	Se intake group						
	Low Se	−2.70 (0.76)	< 0.001	-2.94 (0.88)	0.001	-0.69 (0.65)	0.292
	Moderate Se	0.87 (0.81)	0.279	1.27 (0.70)	0.175	0.14 (0.64)	0.829
	Decline						
ALL INDIVIDUALS	Time	-1.25 (0.07)	< 0.001	−1.25 (0.17)	< 0.001	-1.31 (0.17)	< 0.001
	Slopes (rate of decline)						
	Se intake × Time						
	Low Se × Time			0.11 (0.17)	0.563	0.09 (0.20)	0.650
	Moderate Se			-0.18 (0.21)	0.383	-0.18 (0.21)	0.387
HGS (kg)	Intercept	26.92 (0.96)	< 0.001	26.74 (1.05)	< 0.001	125.81 (59.65)	0.036
	Se intake group						
	Low Se	-1.18 (1.13)	0.296	-0.88 (1.27)	0.488	1.19 (1.19)	0.317
	Moderate Se	-0.34 (1.09)	0.758	-0.19 (1.23)	0.876	0.94 (1.07)	0.384
	Decline						
MEN	Time	−1.76 (0.13)	< 0.001	-1.64 (0.31)	< 0.001	-1.69 (0.31)	< 0.001
	Slopes (rate of decline)						
	Se intake × Time						
	Low Se × Time			-0.19 (0.38)	0.613	-0.25 (0.39)	0.513
	Moderate Se			-0.09 (0.37)	0.799	-0.16 (0.37)	0.669
HGS (kg)	Intercept	14.65 (0.54)	< 0.001	14.71 (0.65)	< 0.001	-23.82 (34.80)	0.045
	Se intake group						
	Low Se	-0.72 (0.58)	0.215	-0.92 (0.18)	0.199	-0.91 (0.69)	0.184
	Moderate Se	0.28 (0.65)	0.666	0.51 (0.80)	0.528	-0.08 (0.73)	0.915
	Decline						
WOMEN	Time	-0.92 (0.07)	< 0.001	-0.95 (0.18)	< 0.001	-1.05 (0.18)	< 0.001
	Slopes (rate of decline)						
	Se intake × Time						
	Low Se × Time			0.10 (0.20)	0.617	0.10 (0.21)	0.626
	Moderate Se			-0.11 (0.23)	0.621	-0.05 (0.23)	0.826

Outcome	Variable	Model 1		Model 2		Model 3	
		**β (SE)**	***p***	**β (SE)**	***p***	**β (SE)**	***p***
TUG (log_{10}-s)	Intercept	1.13 (0.022)	< 0.001	1.13 (0.025)	< 0.001	1.65 (1.08)	0.126
	Se intake group						
	Low Se	0.049 (0.025)	0.048	0.037 (0.028)	0.189	-0.002 (0.024)	0.364
	Moderate Se	0.003 (0.026)	0.917	-0.004 (0.030)	0.884	-0.009 (0.024)	0.722
	Decline						
ALL INDIVIDUALS	Time	0.054 (0.003)	< 0.001	0.049 (0.008)	< 0.001	0.051 (0.010)	< 0.001
	Slopes (rate of decline)						
	Se intake × Time						
	Low Se × Time			0.008 (0.009)	0.383	0.020 (0.012)	0.091
	Moderate Se			0.005 (0.010)	0.623	0.013 (0.013)	0.301
TUG (log_{10}-s)	Intercept	1.067 (0.030)	< 0.001	1.070 (0.033)	< 0.001	-2.949 (3.239)	0.365
	Se intake group						
	Low Se	0.028 (0.036)	0.425	0.017 (0.041)	0.667	-0.016 (0.045)	0.730
MEN	Moderate Se	0.035 (0.035)	0.316	0.037 (0.040)	0.351	0.031(0.041)	0.457
	Decline						
	Time	0.058 (0.005)	< 0.001	0.056 (0.013)	< 0.001	0.054 (0.013)	< 0.001

	Slopes (rate of decline)						
	Se intake × Time						
	Low Se × Time			0.008 (0.015)	0.604	0.002 (0.016)	0.917
	Moderate Se			-0.001 (0.015)	0.921	-0.010 (0.015)	0.507
TUG (log_{10}-s)	Intercept	1.174 (0.031)	< 0.001	1.190 (0.035)	< 0.001	0.629 (2.305)	0.785
	Se intake group						
	Low Se	0.032 (0.033)	0.334	0.015 (0.038)	0.695	-0.023 (0.037)	0.541
	Moderate Se	-0.017 (0.037)	0.655	-0.034 (0.043)	0.431	-0.029 (0.039)	0.462
	Decline						
WOMEN	Time	0.052 (0.004)	< 0.001	0.043 (0.011)	< 0.001	0.042 (0.010)	< 0.001
	Slopes (rate of decline)						
	Se intake × Time						
	Low Se × Time			0.011 (0.012)	0.358	0.006 (0.011)	0.577
	Moderate Se			0.010 (0.013)	0.418	0.003(0.012)	0.801

SE, standard error, Selenium intakes were divided by below LRNI (< 40μg/d) and moderate intakes (41-59, 41-74 μg/d in females and males, respectively). Adequate selenium intakes were used a reference.

Model 1 includes linear trend of time and selenium intakes. Model 2 includes time interaction terms.

Model 3 is adjusted for presence of hand arthritis or use of walking aids, age at baseline, sex, National Statistics Socio-Economic Classification (NS-SEC), self-rated health, energy intake, protein intake, medication use, body mass index (BMI), fat-free mass (FFM), physical activity, cognitive impairment, disability score, misreporters, change in diet. Estimated β coefficients (SE) using HGS and TUG longitudinal data.
